# Isolation and characterization of a new human breast cancer cell line, KPL-4, expressing the Erb B family receptors and interleukin-6

**DOI:** 10.1038/sj.bjc.6690114

**Published:** 1999-02

**Authors:** J Kurebayashi, T Otsuki, M Kurosumi, S Yamamoto, K Tanaka, M Mochizuki, H Nakamura

**Affiliations:** Department of Breast and Thyroid Surgery, Kawasaki Medical School, Kurashiki, Okayama, 701-01, Japan; Department of Hygiene, Kawasaki Medical School, Kurashiki, Okayama, 701-01, Japan; Lombardi Cancer Center, Georgetown University Medical Center, Washington DC, 20007, USA; Department of Pathology, Saitama Cancer Center, Kitaadachi-gun, Saitama, 362, Japan; Yokohama Research Center, Mitsubishi Chemical Corporation, Aoba-ku, Yokohama, 227, Japan

**Keywords:** breast cancer, cell line, Erb B-2, cachexia, interleukin-6

## Abstract

A new human breast cancer cell line, KPL-4, was recently isolated from the malignant pleural effusion of a breast cancer patient with an inflammatory skin metastasis. This cell line can be cultured under serum-free conditions and is tumorigenic in female athymic nude mice. Flow cytometric analysis revealed the expression of Erb B-1, -2 and -3. Dot blot hybridization showed a 15-fold amplification of the *erb*B-2. Reverse transcription-polymerase chain reaction analysis showed a detectable level of mRNA expression of all the Erb B family receptors. In addition, all the receptors were autophosphorylated under a serum-supplemented condition. Unexpectedly, transplanted KPL-4 tumours induced cachexia of recipient mice. A high concentration of interleukin-6 (IL-6) was detected in both the culture medium and the serum of mice. The weight of tumours significantly correlated with the serum IL-6 level. The antiproliferative effect of a humanized anti-Erb B-2 monoclonal antibody, rhuMAbHER2, was investigated. This antibody significantly inhibited the growth of KPL-4 cells in vitro but modestly in vivo. Loss of mouse body weight was partly reversed by rhuMAbHER2. These findings suggest that KPL-4 cells may be useful in the development of new strategies against breast cancer overexpressing the Erb B family receptors and against IL-6-induced cachexia. © 1999 Cancer Research Campaign

Well-characterized human cancer cell lines are essential research resources for studying cancer cell biology as well as for strategies against cancer cell growth and progression. We recently established a new human breast cancer cell line, designated KPL-4, from the malignant pleural effusion of a breast cancer patient with an inflammatory skin metastasis that was resistant to various chemoendocrine therapies and radiation therapy. Inflammatory breast cancer, which is known to show an aggressive clinical behaviour, has been reported to frequently overexpress Erb B-2 or possess an amplified c-*erb* B-2 gene (Charpin et al, 1992; Prost et al, 1994). As expected, immunocytochemical, flow cytometric and reverse transcription polymerase chain reaction (RT-PCR) analyses showed an overexpression of Erb B-2 in this KPL-4 cell line. Dot blot hybridization also showed a 15-fold amplification of the c-*erb* B-2 gene. In addition, Erb B-2 was autophosphorylated under a serum-supplemented condition. These findings suggest that this cell line has highly activated Erb B-2 and may be a useful model for studying the roles of Erb B-2 receptor overexpression in breast cancer cells.

The Erb B family consists of four related proteins. All of these proteins are thought to be growth factor receptors. Recent reports have suggested that these family members are activated by their respective ligands, epidermal growth factor (EGF)/transforming growth factor α (TGF-α) family members or heregulin/neu differentiation factor members through heterodimer formation as well as homodimer formation (Carraway and Cantley, 1994; Earp et al, 1995). It has been suggested that the Erb B-2 signal transduction pathway plays critical roles in growth regulation or the transformation of cells overexpressing multiple Erb B family members (Beerli et al, 1995; Karunagaran et al, 1996; Lewis et al, 1996; Zhang et al, 1996; Graus-Porta et al, 1997). In addition, either epidermal growth factor receptor (EGF-R) or Erb B-2 overexpression in breast cancer has been known to be a worse prognostic indicator (Sainsbury et al, 1987; Wright et al, 1989). A recent report also suggests that Erb B-3 expression is frequently observed in breast tumours of a larger size and higher nuclear grade (Travis et al, 1996). These findings indicate that the Erb B family members and, in particular, Erb B-2 may play an important role in breast cancer cell growth and progression.

Immunohistochemical analysis of the Erb B-2 expression in breast cancer tissues has clearly demonstrated that Erb B-2 overexpression occurs only in breast cancer cells (Walker et al, 1989). Taken together with the importance of the Erb B-2 overexpression in breast cancer progression, breast cancer cell-specific overexpression of Erb B-2 indicates that blockade of the Erb B-2 signal transduction pathway may be a powerful and selective therapeutic method in patients with Erb B-2-overexpressing breast cancer (Shepard et al, 1991; Disis and Cheever, 1997). Recently, promising results from intravenous administration of a humanized anti-Erb B-2 monoclonal antibody to heavily-pretreated patients with Erb B-2-overexpressing recurrent breast cancer have been reported (Baselga et al, 1996).

In this article, the isolation and characterization of our new KPL-4 human breast cancer cell line are presented. In addition, the antiproliferative effect of a humanized anti-Erb B-2 monoclonal antibody, rhuMAbHER2, which is under clinical study, on KPL-4 cells was studied both in vitro and in vivo. rhuMAbHER2 significantly inhibited the growth of KPL-4 cells under both anchorage-dependent and -independent conditions. This antibody also modestly inhibited the growth of KPL-4 cells transplanted into female athymic nude mice. Unexpectedly, the transplanted tumours induced severe cachexia of the recipient mice. Interestingly, a high level of immunoreactive interleukin (IL)-6 was detected in both the culture medium and the serum of the recipient mice.

### MATERIALS AND METHODS

#### Patient and cell culture

A 52-year-old Japanese woman with a primary breast cancer underwent a modified radical mastectomy in September 1992. The histologic diagnosis of the resected specimen was invasive ductal carcinoma with multiple axillary lymph node metastases. Biochemical analyses of estrogen receptor (ER), progesterone receptor (PgR) and EGF-R in the primary tumour showed that this tumour expressed all the three receptors. In spite of adjuvant chemoendocrine therapy with a 5-fluorouracil derivative and tamoxifen, supraclavicular lymph node metastases appeared in November 1994. The patient received a combined therapy including chemoendocrine therapy with medroxyprogesterone acetate, cyclophosphamide, epirubicin and mitomycin C and radiation therapy from November 1994 to December 1995. The recurrent disease progressed with little response to these therapies. Pleural effusion and inflammatory skin metastasis appeared in December 1995. Cytologic examination of the effusion showed atypical epithelial cells. Mitoxantrone was administered into the pleural effusion. Three weeks after the administration, the pleural effusion increased and thoracic drainage was performed to reduce the volume of the effusion. During the same period, the inflammatory skin metastasis progressed and the patient died of breast cancer in February 1996.

Malignant pleural effusion was obtained from the patient at the time of the thoracic drainage. A 50 ml volume of the heparinized fluid was centrifuged at 150 *g* for 10 min. The cell pellet was resuspended and plated in T-25 flasks (Corning Japan, Tokyo, Japan) containing Dulbecco's modified Eagle's medium (DMEM) supplemented with 10% fetal bovine serum (FBS) (ICN Biochemicals, Costa Mesa, CA, USA). Serial passages using 0.05% trypsin and 0.02% EDTA in phosphate-buffered saline (PBS) were performed once or twice a week. Since atypical epithelial cells predominantly grew in culture during all of the passages and cytogenetic analysis as described below strongly indicated the cells to be of a monoclonal origin, we have not attempted to subclone them.

#### Morphologic analysis

Haematoxylin–eosin staining of paraffin-embedded specimens was performed using the conventional method in resected samples from nude mice bearing KPL-4 transplanted tumours. Microphotographs were obtained with an Olympus AH-2 microscope (Olympus, Tokyo, Japan). The cultured cells were observed and phase-contrast microphotographs were taken with an inverted Nikon Diaphot-TMD microscope (Nikon, Tokyo, Japan). For transmission electron microscopy, KPL-4 transplanted tumours were resected, minced into blocks 1 mm in size and fixed with 2.5% glutaraldehyde in PBS for 2 h at 4°C. After washing with PBS, the blocks were post-fixed with 1% osmium tetroxide in 0.1 M cacodylate buffer and embedded in epoxy resin. These blocks were cut into thin sections with a Supernova ultracutter (Reichert-Jung, Vienna, Austria) with a diamond knife, stained with uranyl acetate and lead citrate, and examined with a Hitachi H-7100 electron microscope (Hitachi Electronics, Tokyo, Japan). For immunocytochemical staining, paraffin sections of the tumour samples or cell pellets fixed with 5% buffered-formalin and embedded in paraffin were dewaxed with xylene, hydrated with PBS, treated with hydrogen peroxide for elimination of endogenous peroxidase and then processed by the immunoperoxidase procedure. Rabbit anti-cytokeratin (Milab, Tokyo, Japan), anti-carcinoembryonic antigen (Milab), anti-CA 15-3 (Turner, Tokyo, Japan), anti-vimentin (Dako Japan, Tokyo, Japan) and anti-Erb B-2 (Triton Bioscience, Alameda, CA, USA) antibodies were used as the first antibody. Control experiments were performed by substituting normal rabbit serum for the first antibody. The reaction was visualized by streptavidin–biotin (Nichirei, Tokyo, Japan) techniques following the manufacturer's recommendations (Kurebayashi et al, 1995).

#### Chromosomal analysis

Cytogenetic analysis was performed after this cell line had been passed 16 times. Semi-confluent cells were exposed to 0.1 μg ml$^{-1}$ colcemid for 4 h and detached with a trypsin solution. A hypotonic solution of 0.075 M potassium chloride was added, and the cells were fixed with 3:1 methanol-acetic acid and stained conventionally with Giemsa.

#### Receptor analysis

ER and PgR in the cell pellet and transplanted tumours were measured by an enzyme immunoassay using the ER–EIA and PgR–EIA kits (Dinabot, Tokyo, Japan) following the manufacturer's recommendations. EGF-R in membrane fractions of the samples was determined by a radioreceptor assay using [$^{125}$I]EGF (New England Nuclear, Boston, MA, USA) as the ligand as described elsewhere (Yasui et al, 1988).

#### erb B-2 amplification

Total cellular DNA was extracted by a conventional phenol–chloroform method. DNA dot blot hybridization was performed as described previously (Kurebayashi et al, 1995). In brief, DNA samples were spotted onto Hybond N nylon sheets (Amersham, Arlington Heights, IL, USA), which were hybridized with a $^{32}$P-labelled specific DNA probe and exposed to X-ray films. Hybridization signals were analysed with a BSA2000 bioimaging analyser (Fuji Film, Tokyo, Japan). The degree of oncogene amplification was estimated by comparison with the radioactivity of placental DNA on the same membrane. The actin probe was used as an internal control. The DNA probe was a 1.6 kb *Eco*RI fragment of human c-*erb* B-2 obtained from Otsuka Pharmaceutical Co. (Tokushima, Japan).

### Flow cytometric analysis

Approximately $1 \times 10^6$ cells per sample were harvested with trypsin, stained with first antibodies for 1 h and washed with PBS twice. The samples were then stained with secondary fluorescein isothiocyanate (FITC)-antimouse antibody (Becton Dickinson, San Jose, CA, USA) for 30 min and washed with PBS twice. The level of each Erb B receptor $cell^{-1}$ was analysed by a flow-cytometer (Becton Dickinson). The first antibodies were as follows: anti-EGF-R monoclonal antibody (Oncogene Science, Uniondale, NY, USA), anti-Erb B-2 monoclonal antibody (NeoMarker, Freemont, CA, USA), anti-Erb B-3 monoclonal antibody (NeoMarker) and anti-Erb B-4 monoclonal antibody (NeoMarker).

### RT-PCR analysis

Total cellular RNA was extracted with a TRIzol RNA extraction kit (GIBCO BRL Life Technologies, Gaithersberg, MD, USA) according to the manufacturer's recommendations. One microgram of total RNA and 120 pmol of Oligo (dT) primer in 13 µl was heated to 70°C for 10 min followed by cooling on ice for 1 min. cDNA synthesis was initiated with 200 units of reverse transcriptase (CLONTECH Laboratories Inc., Palo Alto, CA, USA) under conditions recommended by the manufacturer, and the reaction was allowed to proceed at 42°C for 1 h. The reaction was terminated by heating at 94°C for 5 min. cDNA was finally dissolved in a total of 100 µl diethylprocarbonate-treated water and frozen at −20°C before use. Oligonucleotide primers for RT-PCR were designed using published sequences of human EGF-R, Erb B-2, Erb B-3, Erb B-4 and β2-microglobulin and synthesized by the solid-phase triester method (Table 1). PCR was performed using a 1:50 to 1:100 dilution of cDNA, 200 nM of each primer, 200 µM dNTPs, 10 mM Tris-HCl (pH 8.3), 1.5 mM magnesium chloride, 50 mM potassium chloride, 0.01% gelatin and 1 unit of Ampli-*Taq* DNA polymerase (Perkin-Elmer Cetus, Norwark, CT, USA) in a final volume of 20 µl. After an initial denaturation at 94°C for 5 min, annealing at 57°C for 1 min, and extension at 72°C for 1 min were performed on a DNA Thermal Cycler 2400 (Perkin-Elmer Cetus). The final extension was performed for 7 min. PCR products were analysed in a 1.2% agarose gel stained with ethidium bromide.

To compare relative expression levels of each Erb B family receptor in human breast cancer cell lines, the PCR products in the agarose gel were stained with ethidium bromide. A gel image was obtained using the FAS-II UV-image analyser (TOYOBO, Tokyo, Japan), and the densities of the products were quantified using the Quantity One version 2.5 (PDI Inc., Huntington Station, NY, USA). The relative expression levels were calculated as the density of the product of each Erb B family receptor divided by that of β2-microglobulin from the same cell line. The following three human breast cancer cell lines were selected as the positive controls: the MDA-MB 231 cell line, highly expressing EGF-R; the SkBr-3 cell line, highly expressing Erb B-2 and Erb B-3 (Etheir et al, 1996) and the T-47D cell line, highly expressing Erb B-3 and Erb B-4 (Plowman et al, 1993).

### Immunoprecipitation, Western blot and phosphorylation assay

KPL-4 or T-47D cells were cultured in 100 mm-diameter dishes in DMEM supplemented with 5% FBS, and then they were washed twice with PBS and lysed in 1 ml of lysis buffer [Tris-buffered saline (pH 6.8) containing sodium dodecyl sulphate (SDS) and β-mercaptoethanol]. Cell lysates were precleared with 30 µl of normal rabbit serum for 1 h at 4°C. The lysates were then immunoprecipitated with either anti-EGF-R (Oncogene Science), anti-Erb B-2 antibody (NeoMarker), anti-Erb B-3 (C-17, Santa Cruz Biotechnology, Santa Cruz, CA, USA) or anti-Erb B-4 (C-18, Santa Cruz Biotechnology) antibody in combination with protein A agarose (Pharmacia, Piscataway, NJ, USA) overnight at 4°C with gentle agitation. The immunoprecipitates were subsequently washed four times in washing buffer prior to electrophoresis on a 4–16% SDS polyacrylamicle gel electropheresis (SDS-PAGE) (Tris-glycine gradient gel) (Novex, San Diego, CA, USA). Next, they were electroblotted onto Hybond ECL nitrocellulose membranes (Amersham). After being blocked overnight with 5% bovine serum albumin (BSA) in Tris-buffered saline containing 0.05% Tween 20 (pH 7.5), the blots were incubated with monoclonal antiphosphotyrosine antibody (Upstate Biotechnology Inc., Lake Placid, NY, USA) for 1 h. After extensive washing, they were then labelled with peroxidase-tagged sheep antimouse IgG (1:1000) for 30 min and visualized by ECL detection (Amersham) (Tang et al, 1996).

To investigate the cross talk between EGF-R and Erb B-2, 0.1 µg $ml^{-1}$ EGF or 0.1 µg $ml^{-1}$ heregulin β1 (NeoMarker) was added to a routine culture medium 24 h before the cell harvest and then tyrosine phosphorylation of Erb B-2 was explored by the same method as described above.

**Table 1** Sequence of oligonucleotide primers used in RT-PCR and size of predicted products amplified

| Primer (bp) | Sequence | Size of predicted product |
|---|---|---|
| EGF-R | Forward 5′-GCACGAGTAACAAGCTCACG-3′<br>Reverse 5′-TTCCTCTGATGATCTGCAGG-3′ | 231 |
| Erb B-2 | Forward 5′-AGCAGAGGATGGAACACAGC-3′<br>Reverse 5′-CTCCTGGATATTGGCACTGG-3′ | 121 |
| Erb B-3 | Forward 5′-TAGTGGTGAAGGACAATGGC-3′<br>Reverse 5′-AGGAGCACAGATGGTCTTGG-3′ | 116 |
| Erb B-4 | Forward 5′-AGTCAGTGTGTGCAGGAACG-3′<br>Reverse 5′-TCAATGCTGGTTATCTCCAGG-3′ | 130 |
| β 2-microglobulin | Forward 5′-CATCCAGCGTACTCCAAAGA-3′<br>Reverse 5′-GACAAGTCTGAATGCTCCAC-3′ | 165 |

### Cell growth in vitro

Two times $10^5$ cells per well were plated in 12-well plates (SB Medical, Tokyo, Japan) and grown in DMEM supplemented with 5% FBS at 37°C in a 5% carbon dioxide atmosphere. Triplicate wells were trypsinized every other day and the cell number was measured by a Coulter counter (Coulter Electronics, Harpenden, UK). The tumour doubling time was estimated from the linear portion of the growth curve. To investigate the effect of EGF on the growth of KPL-4 cells, $2 \times 10^5$ cells per well were plated in the 12-well plates and grown in DMEM supplemented with 5% FBS for 2 days. Then the cells were washed twice with PBS and grown in a serum-free DMEM supplemented with 0.1–10 μg ml$^{-1}$ EGF. The culture medium was changed every other day. Triplicate wells were trysinized 4 or 8 days after switching the culture medium and the cell number was counted.

The effect of a humanized anti-Erb B-2 monoclonal antibody, rhuMAbHER2 (provided by Mitsubishi Chemical Co., Tokyo, Japan and designated MKC-454 in Japan) was investigated using this system. rhuMAbHER2 is a construct containing the antigen-binding loops of the murine 4D5 antibody with human variable region framework residues plus $IgG_1$ constant domains (Fendley et al, 1990; Carter et al, 1992). Two times $10^5$ KPL-4 cells per well were plated in the 12-well plates and grown in DMEM supplemented with 5% FBS for 2 days. Then the cells were washed twice with PBS and grown in DMEM supplemented with 5% FBS plus 0.1–10 μg ml$^{-1}$ rhuMAbHER2 for 4 days. As the control, concentrations of human $IgG_1$ (Sigma Bioscience, St Louis, MO, USA) of the same amount were added to the medium. The medium was changed every other day. Triplicate wells were trypsinized and the cell number was measured. To investigate the effect of rhuMAbHER2 on the stimulated growth of KPL-4 cells by EGF, 10 μg ml$^{-1}$ rhuMAbHER2 was added to DMEM supplemented with 0.1 μg ml$^{-1}$ EGF.

Anchorage-independent growth in soft agar was also investigated in this cell line. A top layer of 0.25% agarose (Iwaki Chemicals, Tokyo, Japan) in DMEM containing KPL-4 cells at a density of $2 \times 10^4$ per well was added to each well of a 6-well plate (SB Medical) onto a bottom layer of 0.5% agarose in DMEM containing 0.1–10 μg ml$^{-1}$ rhuMAbHER2, 0.1 μg ml$^{-1}$ EGF alone or plus 10 μg ml$^{-1}$ rhuMAbHER2, or 10 μg ml$^{-1}$ human $IgG_1$ (as a control). After 2–7 weeks, colonies were stained with 250 μg per dish 3-(4,5-dimethylthiazol-2-yl)-2,5-diphenyl tetrazolium bromide (Sigma Chemical Co., St Louis, MO, USA) and enumerated using an Omnicon 3600 Tumour Colony Image Analysis System (Imaging Products International Inc., Charlestown, VA, USA).

### Cell growth in vivo

Approximately $1 \times 10^7$ KPL-4 cells were inoculated into the mammary fat pad of 4-week-old female nude mice or severe combined-immunodeficient (SCID) mice (CLEA Japan, Tokyo, Japan). Semi-confluent KPL-4 cells were trypsinized and harvested and viable cells were counted in a haemocytometer using trypan blue exclusion. Tumour volume was calculated as the product of the largest diameter, the orthogonal measurement and the tumour depth. Mean tumour volume was calculated as the sum of tumour volumes divided by the number of tumours. After the mice were sacrificed by cervical dislocation, transplanted tumours, lymph nodes, lungs, liver and kidneys were collected. After measurement of the tumour weight, the resected samples were fixed with 5% buffered-formalin and embedded in paraffin for morphologic analysis.

To investigate the effect of rhuMAbHER2 on the growth of KPL-4 cells transplanted into nude mice, approximately $5 \times 10^6$ KPL-4 cells were injected into the right and left mammary fat pads of 4-week-old female nude mice (two sites per mouse). In addition, to investigate the effect of rhuMAbHER2 on the in vivo growth of KPL-1 human breast cancer cells that do not overexpress Erb B-2 (as the control cells), the cells were injected into 4-week-old female nude mice in the same manner. In the rhuMAbHER2-treated group, 20 mg kg$^{-1}$ rhuMAbHER2 was intraperitoneally administered as a loading dose 2 weeks after the cell injections and then 10 mg kg$^{-1}$ rhuMAbHER2 was intraperitoneally administered once every week during 3–5 weeks after the cell injections. The same volumes of human $IgG_1$ were administered in the control group in the same manner. Five mice (ten tumours) were treated in each group. Three-dimensional tumour size was measured with calipers every week after the cell injections. The body weight of the mice was measured every week before the administration of rhuMAbHER2. All the mice were sacrificed 5 weeks after the cell injections. Then, mouse blood was collected and the serum was stored at −80°C before use. Transplanted tumours, axillary lymph nodes and lungs were resected, and the weight of tumours was measured.

The animal protocols for these experiments were approved by the Animal Care and Use Committee of Kawasaki Medical School.

### Measurement of IL-6

IL-6 concentrations in the cultured medium and mouse serum were measured with a chemiluminescent enzyme immunoassay kit (Fujirebio, Tokyo, Japan) according to the manufacturer's recommendations. Briefly, a mouse anti-human IL-6 monoclonal antibody (HH 61-10) was used as the first antibody and a mouse anti-human IL-6 monoclonal antibody (Fujirebio) labelled with alkaline phosphatase (HH 61-2 Fab′) was used as the second one. After removing the unbound second antibody, 3-(2′-spiroadmantane)-4-methoxy-4-(3″-phosphoryloxy)phenyl-1,2-dioxetane disodium salt was added. Chemiluminescence was measured with a Lumipulse luminometer (Fujirebio). As the standard, 20-1000 pg ml$^{-1}$ human recombinant IL-6 was used. The minimal detectable concentration of IL-6 was 0.2 pg ml$^{-1}$. The intra-assay coefficients of variation for the high, middle and low sample levels were 2.8%, 2.2% and 3.8%, respectively. The inter-assay coefficients of variation for these sample levels were 3.6%, 4.9% and 8.6% (Takemura et al, 1996).

### Statistical analysis

All data are expressed as the mean ± SD. Analysis of variance (ANOVA) with StatView computer software (ATMS Co., Tokyo, Japan) was used to compare differences in the tumour volume, tumour weight, mouse body weight and IL-6 concentration between two different groups. Two-sided $P < 0.05$ was considered as statistically significant. The correlation coefficients were calculated by linear regression analysis with the same software.

## RESULTS

### Isolation and characterization of the KPL-4 cell line

KPL-4 cells were isolated from the malignant pleural effusion of a postmenopausal breast cancer patient with an inflammatory skin

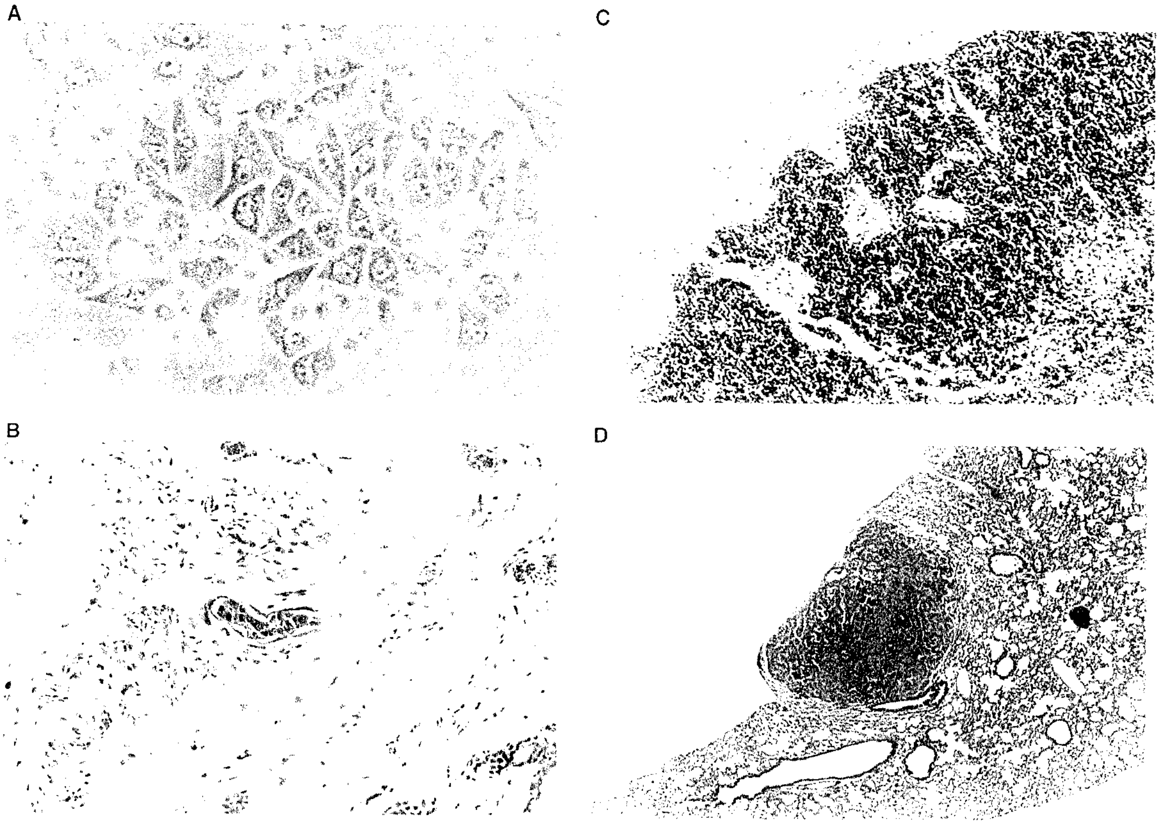

Figure 1 Microphotographs of KPL-4 cells in vitro and of KPL-4 transplanted tumours and their metastases in nude mice. (A) KPL-4 cells growing in a monolayer fashion (a phase-contrast photograph, original × 100). (B) The tumour occasionally showing vascular invasion into the surrounding stromal tissues (haematoxylin-eosin staining, original × 150). (C) Tumour cells in the marginal sinus of an axillary lymph node (haematoxylin-eosin staining, original × 75). (D) A metastasis in the lung (haematoxylin-eosin staining, original × 30)

metastasis which was resistant to various therapies. Collected cells from the patient were cultured in DMEM supplemented with 5% FBS through over 70 passages for over a year. Although KPL-4 cells basically grow in a monolayer fashion like cobblestones, they tend to pile up on each other when they reach confluency. Each cell is polygonal and possesses a large nucleus with a single prominent nucleolus (Figure 1A). The population doubling time of this cell line in DMEM supplemented with 5% FBS is approximately 30 h. Interestingly, KPL-4 cells grow slowly under serum-free conditions, and exogenous addition of EGF (0.1–10 $\mu g\ ml^{-1}$) dose-dependently stimulated their growth (Figure 2).

Karyotype analysis of the KPL-4 cell line at the 16th passage revealed the median chromosome number to be 53 with a range from 52 to 54 ($n$ = 40). G-banding indicated marked chromosomal abnormalities (Figure 3). Sixteen common chromosomal aberrations were observed in all ten metaphases explored. Immunocytochemical analysis showed that KPL-4 cells express cytokeratin, carcinoembryonic antigen, CA 15-3 and Erb B-2, but not vimentin and ER. Biochemical analysis indicated the absence of ER and PgR in this cell line. These findings were observed in both the culture cells and the transplanted tumours. Electron microscopic analysis revealed intracytoplasmic lumen and desmosome structures in the KPL-4 cells transplanted into nude mice. These findings strongly suggest that this cell line is derived from a monoclonal human breast cancer cell.

To investigate the tumorigenicity of this cell line, approximately $1 \times 10^7$ KPL-4 cells were injected into the mammary fat pad of female nude mice or SCID mice. KPL-4 cells developed fast-growing tumours at a take rate of 100% in both nude mice and SCID mice ($n$ = 10 each), and the growth curves of KPL-4 transplanted tumours in these mice were identical (Figure 4). The mice bearing KPL-4 tumours became cachexic 3 weeks after the cell injections and 20% of them died within 5 weeks after the cell injections in two separate experiments. Postmortem examination revealed that the transplanted tumours were occasionally invasive to surrounding tissues (Figure 1B), such as skin and muscles, and a massive central necrosis of the tumours was frequently observed. No macroscopic metastasis was observed in the lymph nodes, lungs, liver or kidneys. However, microscopic examination of the resected specimens showed metastases into the lymph nodes (Figure 1C) and lungs (Figure 1D).

### Erb B family receptor expression in KPL-4 cells

Immunocytochemical analysis of the KPL-4 cells revealed over-express Erb B-2 oncoprotein. A radio-receptor assay showed that these cells possess approximately 50 fmol per mg protein of EGF-R in the membrane fraction. Dot blot hybridization indicated an approximately 15-fold amplification of the *erb* B-2 gene in these cells (Figure 5A). These findings were observed in both the culture cells and the transplanted tumours. Flow cytometric analysis using monoclonal antibodies against Erb B family receptors demonstrated a high level of Erb B-2 and Erb B-3, a low level of EGF-R and no expression of Erb B-4 (Figure 5B). However, an RT-PCR

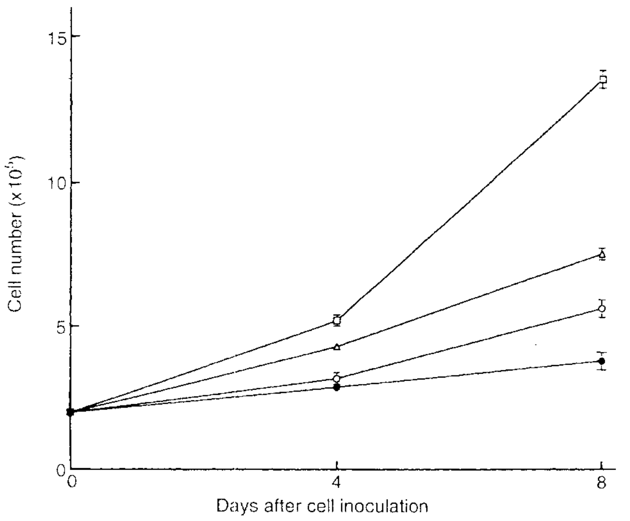

**Figure 2** Anchorage-dependent growth of KPL-4 cells in a serum-free medium supplemented with EGF. KPL-4 cells were incubated with D-MEM with 0 (●), 0.1 (○), 1.0 (△) or 10 (□) μg ml$^{-1}$ EGF for 4 or 8 days. Bars show SD

analysis detected the mRNA of all the Erb B family receptors in KPL-4 cells. The amount of PCR products for Erb B-2 and Erb B-4 was higher than that for EGF-R and Erb B-3 (Figure 5C). To compare relative expression of each Erb B family receptor, three human breast cancer cell lines were also analysed by the same RT-PCR method. As shown in Table 2, the relative expression level of Erb B-2 in KPL-4 cell line was highest in the explored cell lines. Immunoprecipitations were also performed using specific anti-EGF-R/Erb-2/Erb B-3/Erb B-4 antibodies, followed by Western blotting analysis with an anti-phosphotyrosine antibody. A strong tyrosine phosphorylation of all four Erb B family receptors was observed in KPL-4 cells under a serum-supplemented condition but not in T-47D cells (Figure 5D). These findings suggest that KPL-4 cells possess all the Erb B family receptors activatable under a serum-supplemented condition.

### rhuMAbHER2 inhibits the growth of KPL-4 cells

The addition of 0.1–10 μg ml$^{-1}$ rhuMAbHER2 to the medium supplemented with 5% FBS significantly inhibited the anchorage-dependent growth of KPL-4 cells (Figure 6A). However, the same concentrations of rhuMAbHER2 more clearly inhibited the anchorage-independent growth of KPL-4 cells in a serum-supplemented medium (Figure 6B). Furthermore, the addition of 10 μg ml$^{-1}$ rhuMAbHER2 to a serum-free medium supplemented with 0.1 μg ml$^{-1}$ EGF significantly inhibited the stimulated growth of KPL-4 cells by EGF (Figure 6C). More growth inhibition was observed under an anchorage-independent condition (Figure 6D).

To clarify the cross-talk between EGF-R and Erb B-2, tyrosine phosphorylation of Erb B-2 induced by EGF or heregulin was investigated. As expected in comparison with the control, Erb B-2 phosphorylation was enhanced by the exogenous addition of 0.1 μg ml$^{-1}$ EGF into the medium (Figure 7).

To investigate the antiproliferative effect of rhuMAbHER2 in vivo rhuMAbHER2 was administered intraperitoneally to mice bearing KPL-4 transplanted tumours once every week for 4 weeks. The tumour volume 5 weeks after the cell inoculations was 826.6 ± 295.3 mm$^3$ ($n$ = 10) in the rhuMAbHER2-treated group and 1055.7 ± 542.9 mm$^3$ ($n$ = 10) in the control group (Figure 8A). The tumour weight was 0.67 ± 0.17 g ($n$ = 10) in the rhuMAbHER2-treated group and 0.91 ± 0.30 g ($n$ = 10) in the control group. rhuMAbHER2 modestly inhibited the growth of KPL-4-transplanted tumours ($P$ = 0.256 for the tumour volume and $P$ = 0.036 for tumour weight). Because nude mice bearing KPL-4 tumours became cachexic 3 weeks after the cell inoculations, mouse body weight was compared between the two groups. The mouse body weight in the treated group (19.5 ± 1.1 g) tended to be higher than

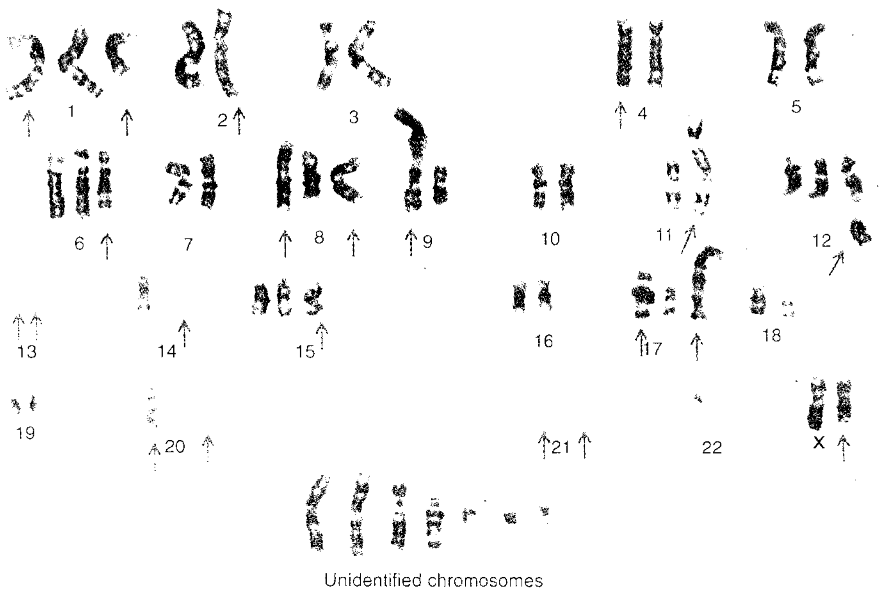

**Figure 3** Karyotype analysis of KPL-4 cells displaying marked chromosomal abnormalities and unidentified marker chromosomes. Arrows indicate abnormal chromosomes

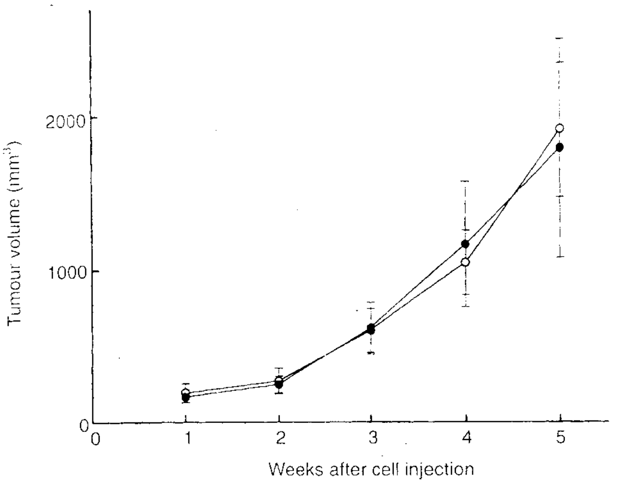

**Figure 4** In vivo growth of KPL-4 cells transplanted into female athymic nude mice (●) or into female SCID mice (○). One times $10^7$ KPL-4 cells were injected into the mammary fat pad of respective mice. Bars show SD

that (17.3 ± 2.1 g) in the control group 4 weeks after the cell inoculations ($P$ = 0.086, Figure 8B). Histologic examination of the resected tumours revealed no remarkable differences in tumour cell morphology or in lymphocyte infiltration between the two groups. The same treatment with rhuMAbHER2 influenced neither the growth of KPL-1-transplanted tumours (data not shown) nor the mouse body weight (Figure 8B). The body weight of the mice bearing KPL-1 tumours was significantly higher than that of mice bearing KPL-4 tumours ($P < 0.01$, Figure 8B).

### IL-6 levels in the culture medium and mouse serum

The concentrations of IL-6 in the culture medium and the mouse serum were measured with a chemiluminescent enzyme assay. The basal secretion of IL-6 from KPL-4 cells into DMEM supplemented with 5% FBS was approximately 6.4 pg per 48 h per one million cells. The concentration of IL-6 in the culture medium of KPL-1 cells was below 0.2 pg ml⁻¹. Interestingly, a significant correlation was found between the concentrations of IL-6 in the

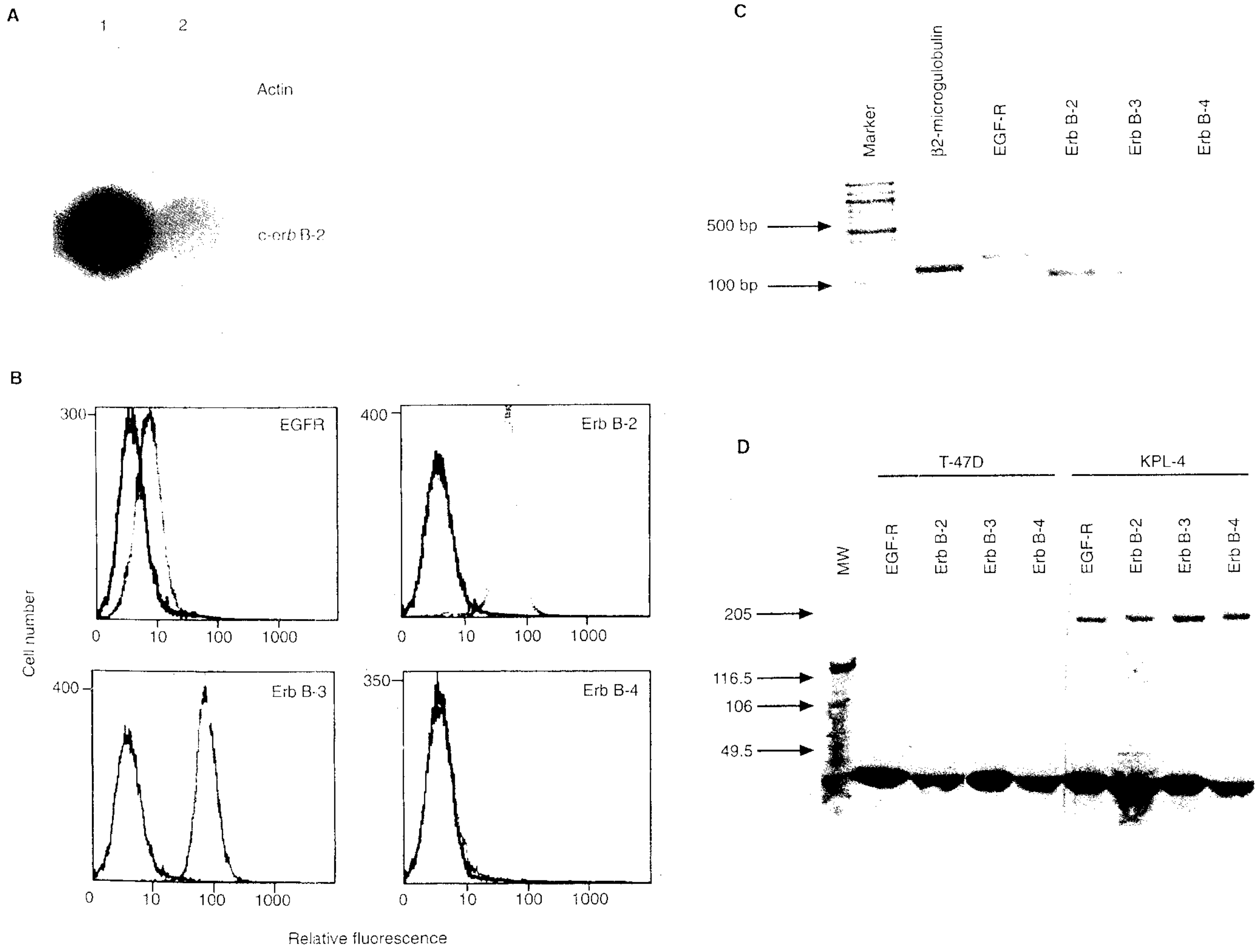

**Figure 5** (**A**) DNA dot blot hybridization of the *c-erb* B-2 gene in KPL-4 cells; Lane 1, KPL-4 cells; Lane 2, human placental DNA. Actin was used as an internal control. (**B**) Flow cytometric analysis of the Erb B family receptors in KPL-4 cells. (▬), incubated without a first antibody. (–), incubated with a first antibody against each Erb B family receptor. (**C**) RT-PCR analysis of the Erb B family receptors in KPL-4 cells. The gel images are shown in inverted presentation. (**D**) Autophosphorylation of the Erb B family receptors in KPL-4 and T-47D cells. Cell lysate was subjected to immunoprecipitation with a specific antibody against each receptor. The precipitates were then subjected to Western blotting with an antiphosphotyrosine antibody. Constitutive phosphorylation of all the Erb B family receptors was observed only in KPL-4 cells.

Table 2 Relative expression levels of Erb B family receptors in human breast cancer cell lines

| Cell line | Relative expression[a] EGF-R | Erb B-2 | Erb B-3 | Erb B-4 |
|---|---|---|---|---|
| KPL-4 | 0.56 | 0.83 | 0.39 | 0.51 |
| MDA-MB 231 | 0.65 | 0.52 | 0.13 | 0.18 |
| SkBr-3 | 0.28 | 0.78 | 0.59 | 0.75 |
| T-47D | 0.29 | 0.74 | 0.59 | 0.98 |

[a]PCR-products in agarose gel were stained with ethidium bromide, a gel image was obtained using an image analyser, and the densities of the products were quantified by a computer-assisted method. The relative expression levels were calculated as the density of the product of each Erb B family receptor divided by that of β2-microglobulin from the same cell line. Each value represents the mean of more than two separate experiments.

mouse sera and the KPL-4 tumour weights ($r$ [correlation coefficient] = 0.831, $n = 10$, $P < 0.01$) (Figure 9). In addition, the serum concentration of IL-6 in the rhuMAbHER2-treated group (38.2 ± 9.3 pg ml$^{-1}$, $n = 5$) was significantly lower than that in the control group (67.0 ± 26.1, $n = 5$) ($P < 0.05$).

### DISCUSSION

Various human breast cancer cell lines have been isolated worldwide and used in studies to understand breast cancer cell biology and in developing new strategies against breast cancer cell growth and progression. Two human breast cancer cell lines have already been isolated in our laboratory and their characterization has been reported elsewhere (Kurebayashi et al, 1995, 1996). In this paper, the isolation and characterization of a third human breast cancer cell line, KPL-4, established in our laboratory are presented. This cell line was derived from a patient with a very aggressive inflammatory skin metastasis. As expected, KPL-4 cells grew more rapidly than the other two cell lines both in vitro and in vivo. Our preliminary characterization revealed expression of all four Erb B family receptors in this cell line. In particular, both overexpression of Erb B-2 protein and *erb* B-2 gene amplification were observed in this cell line. Interestingly, KPL-4 cells can grow under a serum-free condition and also can grow rapidly in female athymic nude mice or SCID mice. There have been only two reports indicating that human breast cancer cell lines overexpressing Erb B-2 can produce tumours in nude mice. In one of these reports, the 21MT cell line and its subclones produced transplanted tumours in nude mice after 2–13 weeks latency (Band et al, 1989). In the other report, the BT-474 cell line produced transplanted tumours in nude mice that received implants of slow release oestrogen pellets (Baselga et al, 1998). Therefore, to the best of our knowledge, this KPL-4 cell line is the first human breast cancer cell line that overexpresses Erb B-2 and can produce rapid-growing tumours without oestrogen supplementation in vivo. In addition, it should be noted that transplanted KPL-4 tumours induce severe cachexia in recipient mice and begin to kill the mice only 4 weeks after cell inoculations. Postmortem histologic examination revealed metastases from the transplanted tumours into lymph nodes and lungs

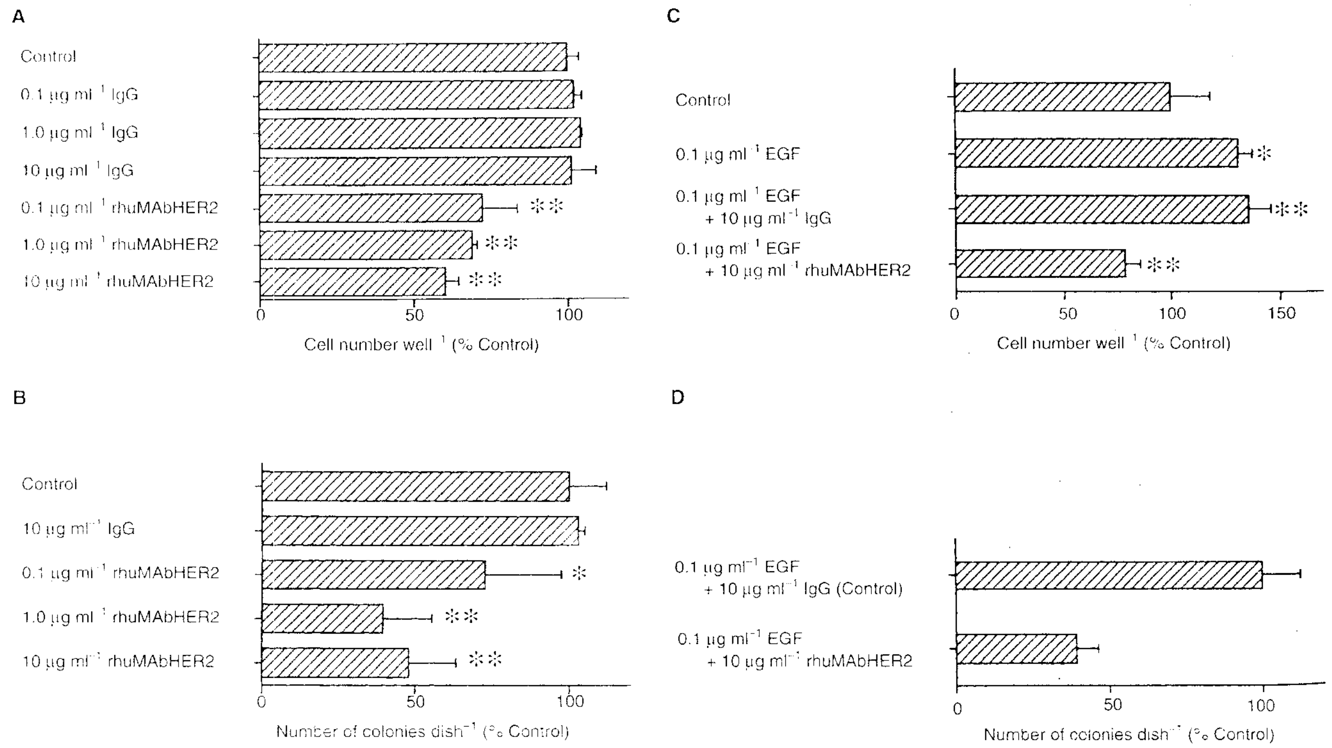

**Figure 6** Antiproliferative effect of rhuMAbHER2 on KPL-4 cells in vitro. (**A**) Anchorage-dependent growth in DMEM supplemented with 5% FBS; 0.1–10 μg ml⁻¹ rhuMAbHER2 significantly inhibited the growth. (**B**) Anchorage-independent growth in DMEM supplemented with 10% FBS; 1.0–10 μg ml⁻¹ rhuMAbHER2 significantly inhibited the growth. (**C**) Anchorage-dependent growth under serum-free conditions. Although 0.1 μg ml⁻¹ EGF significantly stimulated the growth, 10 μg ml⁻¹ rhuMAbHER2 alone did not; 10 μg ml⁻¹ rhuMAbHER2 significantly inhibited the stimulated growth by 0.1 μg ml⁻¹ EGF ($P < 0.01$). (**D**) Anchorage-independent growth in DMEM supplemented with 2% FBS plus 0.1 μg ml⁻¹ EGF; 10 μg ml⁻¹ rhuMAbHER2 significantly inhibited the stimulated growth by 0.1 μg ml⁻¹ EGF ($P < 0.01$). Bars show SD. **, $P < 0.01$; *, $P < 0.05$ in comparison with control except for Figure 6C and D

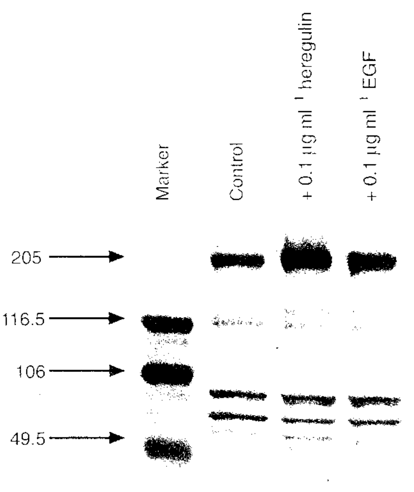

Figure 7 Tyrosine phosphorylation induced by the exogenous addition of EGF (0.1 μg ml$^{-1}$) and heregulin β1 (0.1 μg ml$^{-1}$) to the culture medium. After 24-h treatment, the cell lysate was subjected to immunoprecipitation with a specific antibody against Erb B-2. The precipitates were then subjected to Western blotting with an antiphosphotyrosine antibody. Tyrosine phosphorylation was enhanced by EGF as well as by heregulin β1 in comparison with control.

(Figure 1). These findings indicate that KPL-4 cells possess a very aggressive phenotype, like that of an inflammatory breast cancer, and that this cell line may be a useful model in studying the biology of such an aggressive breast cancer.

Although RT-PCR analysis showed detectable expression of all four Erb B family receptors (Figure 5C) and immunoprecipitation using specific antibody against each Erb B family receptor followed by Western blot analysis with antiphosphotyrosine antibody showed a remarkable autophosphorylation of each Erb B family receptor under a serum-supplemented condition (Figure 5D), flow cytometric analysis of the expression of Erb B family receptors on the cell surface revealed no detectable expression of Erb B-4 (Figure 5B). This discrepancy may be explained as follows. First, it may be possible that the antibody used in the immunoprecipitation was more sensitive than the antibody used in the flow cytometric analysis. The former recognized the intracellular domain of Erb B-4 and the latter recognized its extracellular domain. It might be possible that the latter could not detect the low expression of Erb B-4 in KPL-4 cells. The second possibility is that Erb B-4 may be internalized under a serum-supplemented condition and the antibody raised against the extracellular domain of Erb B-4 could not detect Erb B-4 expression in the flow cytometric analysis. Precise causes of this discrepancy remain to be elucidated. However, a remarkable autophophorylation of each Erb B family receptor strongly suggests that all of the Erb B family receptors are activated under a routine culture condition and that they may participate in the growth of KPL-4 cells. It is conceivable that some of the ligands for the Erb B family receptors may be produced by KPL-4 cells and may induce autophosphorylation of the Erb B family receptors in an autocrine or paracrine manner. In our preliminary experiment using a semi-quantitative RT-PCR, mRNA expression of the heregulin family members was undetectable in KPL-4 cells (unpublished data). Further studies are needed to clarify this interesting hypothesis.

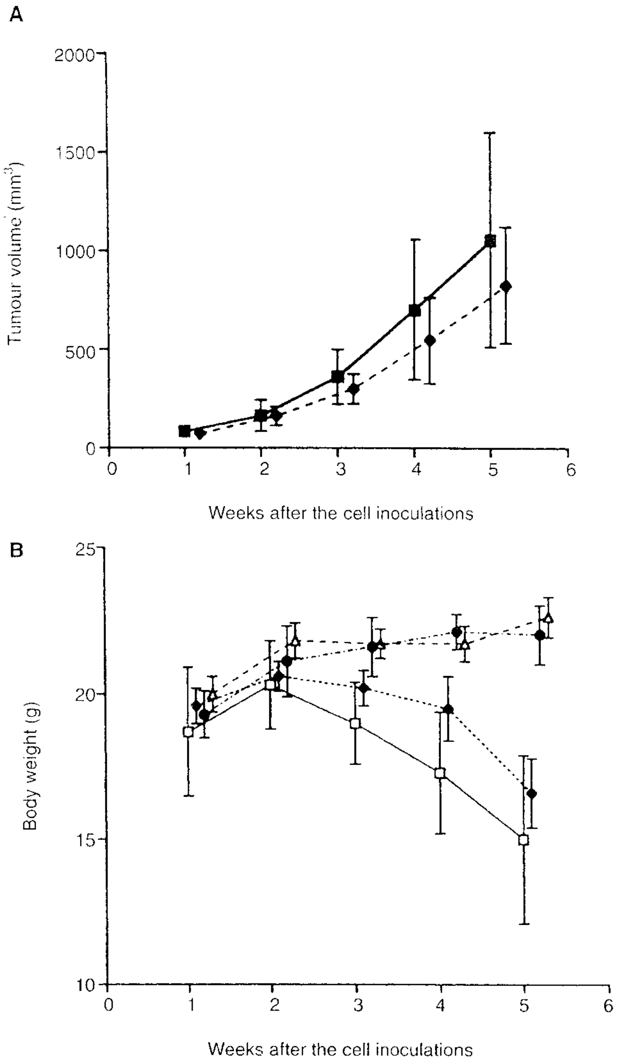

Figure 8 (A) Antiproliferative effect of rhuMAbHER2 on KPL-4-transplanted tumours in female athymic nude mice ($n$ = 10 in each group). Five times $10^6$ KPL-4 cells were injected into the mammary fat pad of nude mice. rhuMAbHER2 was administered once a week from 2 weeks after the cell injections. ■, control group; ◆, rhuMAbHER2-treated group. Bars show SD. (B) Changes in mouse body weight ($n$ = 5 in each group). △, control group (injected with KPL-1 cells); ●, rhuMAbHER2-treated group (injected with KPL-1 cells); □, control group (injected with KPL-4 cells); ◆ rhuMAbHER2-treated group (injected with KPL-4 cells). Bars show SD

Our preliminary study indicated that cachexia in this nude mouse model may be induced by the secretion of IL-6 from KPL-4 cells. A high concentration of immunoreactive IL-6 was detected in the culture medium of KPL-4 cells and in the serum of mice bearing KPL-4-transplanted tumours. In addition, the concentrations of IL-6 in the serum significantly correlated with the weight of KPL-4-transplanted tumours (Figure 8). Very low levels of IL-6 were detected in the culture medium of KPL-1 cells (as a control) and the serum of mice bearing KPL-1 tumours (data not shown). A recent report suggest that oesophageal cancer patients with serum IL-6 levels equal to, or higher than, 7 pg ml$^{-1}$ are more cachexigenic than those with levels lower than 7 pg ml$^{-1}$ (Oka et al. 1996). The serum levels of IL-6 in nude mice transplanted with KPL-4 cells were higher than 25 pg ml$^{-1}$ (Figure 8). Another recent report suggests that the serum IL-6 level of patients with metastatic breast cancer is

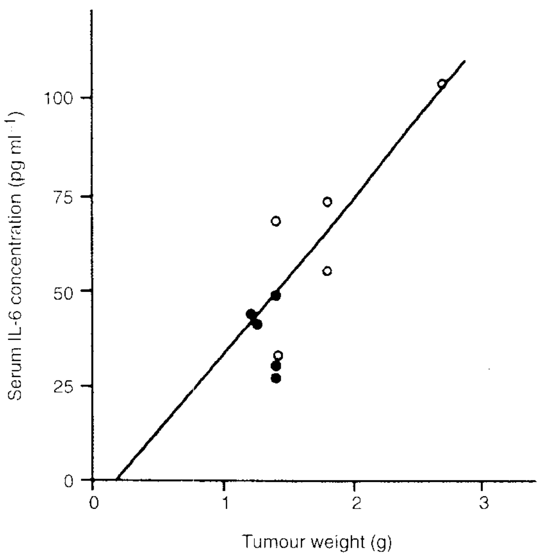

**Figure 9** Relationship between the weight of KPL-4-transplanted tumours and the IL-6 concentration in the serum of recipient mice. IL-6 concentrations were measured with a chemiluminescent enzyme immunoassay. Correlation coefficient ($r$) = 0.831 ($n$ = 10, $P < 0.01$). ○, control group; ●, rhuMAbHER2-treated group significantly higher than that for normal individuals and that the decrease in serum IL-6 levels in those patients significantly correlates with their weight gain (Yamashita et al, 1996). These findings suggest that overproduction and secretion of IL-6 from breast cancer cells is one of the main causes of cancer-induced cachexia in breast cancer. This hypothesis has been supported by experimental studies using a mouse colon carcinoma cell line, colon 26, and its subclones (Strassmann et al, 1992; Ohe et al, 1993; Fujimoto-Ouchi et al, 1995; Yasumoto et al, 1995). In our preliminary experiment, other possible cachectic factors, such as tumour necrosis factor-α, leukaemia inhibitory factor, interferon-γ and IL-1 were not detectable in the serum of mice bearing KPL-4-transplanted tumours (unpublished data). Further investigation is needed to clarify whether IL-6 is the sole cause of cachexia in this model system.

A humanized monoclonal antibody, rhuMAbHER2, was examined in this study because it has recently been used in a clinical Phase I trial in Japan. rhuMAbHER2 has already been evaluated in a Phase II clinical trial in the USA in patients with metastatic breast cancers that overexpress Erb B-2 oncoprotein (Baselga et al, 1996). As was expected, this antibody significantly inhibited the growth of KPL-4 cells in vitro and modestly in vivo. Because of the poor tumorigenicity of human breast cancer cell lines overexpressing Erb B-2, only one report describing the anti-tumour effect of this antibody has been published so far (Baselga et al, 1998). The antiproliferative effect of this antibody in vivo was modest and the inhibition rate was approximately 30%. However, it should be noted that this antibody could inhibit the Erb B-2 signalling pathway in the transplanted tumours but could not induce antibody-dependent cell-mediated cytolysis in this immunocompromised nude mouse model. Thus, this antibody could be expected to be more effective in women with normal immunity. Interestingly, the administration of this antibody partly reversed cachexia in recipient mice. The results of our preliminary experiment using this KPL-4 cell line suggest that rhuMAbHER2 does not influence IL-6 secretion from KPL-4 cells in vitro (unpublished data). Thus, it is likely that the antiproliferative effect of this antibody resulted in reduction of tumour volumes, in decrease of total IL-6 production from the tumours and in amelioration of mouse cachexia.

rhuMAbHER2 significantly inhibited the stimulated growth of KPL-4 cells by EGF (Figure 6C, D). EGF dose-dependently stimulated the growth of KPL-4 cells under a serum-free condition (Figure 2). Furthermore, our preliminary study indicates that EGF stimulates the autophosphorylation of the Erb B-2 receptor in KPL-4 cells (Figure 7). It has been suggested that EGF-R prefers heterodimerization with Erb B-2 to homodimerization with EGF-R (Wada et al, 1990; Quian et al, 1994; Earp et al, 1995). These findings suggest that EGF-stimulated heterodimerizaton between EGF-R and Erb B-2, and the subsequent transactivation of the Erb B-2 signalling pathway, may take part in the growth-promoting effect of EGF in this cell line. In other words, the blockade of the Erb B-2 signalling pathway may, at least in part, inhibit the EGF-stimulated growth of breast cancer cells expressing both EGF-R and Erb B-2. The effect of rhuMAbHER2 on EGF-stimulated transactivation of the Erb B-2 protein is under investigation. Recently, some authors have observed that EGF can activate the heterodimer of Erb B-2 and Erb B-3 in the absence of EGF-R in murine IL-3-dependent 32D myeloid progenitor cells transfected with Erb B-2 and Erb B-3 and in MDA-MB 134 human breast carcinoma cells (Alimandi et al, 1997). Thus, it might be possible that rhuMAbHER2 inhibits Erb B-2 signal transduction through not only heterodimerization between EGF-R and Erb B-2 but also heterodimerization between Erb B-2 and Erb B-3. Further experiments are needed to elucidate the action mechanisms of the antiproliferative effect of rhuMAbHER2 on breast cancer cells overexpressing multiple Erb B family receptors.

In conclusion, we have established a new human breast cancer cell line, KPL-4, from a recurrent breast cancer patient. This cell line expresses all four members of the Erb B family but, in particular, Erb B-2. KPL-4 cells can grow rapidly in culture and form tumours in female nude mice. Transplanted tumours caused severe cachexia in mice and metastasized into the lymph nodes and lungs. A high level of immunoreactive IL-6 was detected in the culture medium and the serum of mice bearing KPL-4-transplanted tumours. A humanized anti-Erb B-2 monoclonal antibody, rhuMAbHER2, inhibited the growth of KPL-4 cells both in vitro and in vivo. In addition, this antibody significantly decreased serum IL-6 levels of recipient mice and partly reversed mouse body weight loss. These findings indicate that this newly-established cell line, KPL-4, may be a useful model for studying the cell biology of aggressive breast cancers overexpressing the Erb B family receptors and IL-6 and for investigating a novel therapeutic strategy against them.

### ACKNOWLEDGEMENTS

This work was supported in part by a grant from the Ministry of Education, Science, Sports and Culture of Japan and by the research project grants (No. 8-301, 9-106) from Kawasaki Medical School. The authors would like to thank Dr Robert B Dickson, Lombardi Cancer Center, Georgetown University, for his helpful comments on this manuscript.
